# Effect of Y Content on Precipitation Behavior, Oxidation and Mechanical Properties of As-Cast High-Temperature Titanium Alloys

**DOI:** 10.3390/ma16134784

**Published:** 2023-07-02

**Authors:** Jiafeng Shen, Binguo Fu, Yufeng Wang, Tianshun Dong, Jingkun Li, Guolu Li, Jinhai Liu

**Affiliations:** 1 Key Laboratory for New Type of Functional Materials in Hebei Province, School of Materials Science and Engineering, Hebei University of Technology, Tianjin 300401, China; shenjf_1998@163.com (J.S.); dongtianshun111@163.com (T.D.); tjjk_zy@126.com (J.L.); liguolu@hebut.edu.cn (G.L.); jhliu57@126.com (J.L.); 2 Aerospace Product Center, Tianjin Institute of Aerospace Mechanical and Electrical Equipment, Tianjin 300301, China; wangyufeng_412@163.com

**Keywords:** high-temperature titanium alloy, Y content, precipitated phases, oxidation behavior, mechanical property

## Abstract

To improve the heat resistance of titanium alloys, the effects of Y content on the precipitation behavior, oxidation resistance and high-temperature mechanical properties of as-cast Ti-5Al-2.75Sn-3Zr-1.5Mo-0.45Si-1W-2Nb-xY (x = 0.1, 0.2, 0.4) alloys were systematically investigated. The microstructures, phase evolution and oxidation scales were characterized by XRD, Laser Raman, XPS, SEM and TEM. The properties were studied by cyclic oxidation as well as room- and high-temperature tensile testing. The results show that the microstructures of the alloys are of the widmanstätten structure with typical basket weave features, and the prior β grain size and α lamellar spacing are refined with the increase of Y content. The precipitates in the alloys mainly include Y_2_O_3_ and (TiZr)_6_Si_3_ silicide phases. The Y_2_O_3_ phase has specific orientation relationships with the α-Ti phase: (002)_Y_2_O_3__ // ($\bar{1}$ $\bar{1}$20)_α-Ti_, [110]_Y_2_O_3__ // [$\bar{4}$401]_α-Ti_. (TiZr)_6_Si_3_ has an orientation relationship with the β-Ti phase: (02$\bar{2}$$\bar{1}$)_(TiZr)_6_Si_3__ // (011)_β-Ti_, [$\bar{1}$2$\bar{1}$6]_(TiZr)_6_Si_3__ // [04$\bar{4}$]_β-Ti_. The 0.1 wt.% Y composition alloy has the best high-temperature oxidation resistance at different temperatures. The oxidation behaviors of the alloys follow the linear-parabolic law, and the oxidation products of the alloys are composed of rutile-TiO_2_, anatase-TiO_2_, Y_2_O_3_ and Al_2_O_3_. The room-temperature and 700 °C UTS of the alloys decreases first and then increases with the increase of Y content; the 0.1 wt.% Y composition alloy has the best room-temperature mechanical properties with a UTS of 1012 MPa and elongation of 1.0%. The 700 °C UTS and elongation of the alloy with 0.1 wt.% Y is 694 MPa and 9.8%, showing an optimal comprehensive performance. The UTS and elongation of the alloys at 750 °C increase first and then decrease with the increase of Y content. The optimal UTS and elongation of the alloy is 556 MPa and 10.1% obtained in 0.2 wt.% Y composition alloy. The cleavage and dimples fractures are the primary fracture mode for the room- and high-temperature tensile fracture, respectively.

## 1. Introduction

High-temperature titanium alloys are widely used in aerospace and military fields due to their excellent properties, such as low density, high specific strength, high specific stiffness and excellent high-temperature creep properties [1,2,3]. With the development of aerospace vehicles towards higher flight speeds, in addition to requiring titanium alloys with low density and high strength, they are also required to withstand higher temperatures [4,5]. At present, the maximum service temperature of titanium alloys used for a long period can reach 600 °C. Typical brands are Ti-1100, IMI834, BT36, Ti60 and Ti600 alloys [6,7]. However, when an aerospace vehicle flies at supersonic speeds, it has strong friction with the air, generating extremely high heat. The surface material temperature can be maintained at 700 °C for a short period of time, which may have a serious impact on the structural integrity of the aircraft [8]. Therefore, there is an urgent need to develop 700 °C short-term high-temperature titanium alloy for manufacturing high-temperature components of aircraft. Fully utilizing the matching effect of various alloy elements is one of the important methods for developing and improving the microstructure and properties of cast high-temperature titanium alloys. Alloying elements in titanium alloys are usually classified into α or β stabilizing additions depending on whether they increase or decrease the α/β transformation temperature of pure Ti. Currently, the composition design for high-temperature titanium alloys with temperatures up to 600 °C is the Ti-Al-Sn-Zr-Mo-Si series [9,10]. Some studies have suggested that the addition of β-stabilizer elements W and Nb can further increase its usage temperature [11,12,13]; however, the increase in service temperature is limited.

The rare earth element Y has strong chemical activity and deoxidation, which can purify the titanium alloy matrix and improve the high-temperature mechanical properties and oxidation resistance of the alloy. Y mainly affects the microstructures of titanium alloys by forming the Y_2_O_3_ phase, thereby improving their strength and toughness [14,15,16]. Previous studies have pointed out that the addition of rare earth elements to a Ti-Al-Sn-Zr-Mo-Si series titanium alloy could refine the oxide particles and improve the oxidation resistance of the alloy [17]. Therefore, the addition of the Y element to the high-temperature titanium alloys may be an effective method to improve the heat resistance. However, the precipitation behavior of the Y_2_O_3_ phase, as well as the interaction between Y and other alloy elements and the mechanism of their impact on the high-temperature properties of the alloys are still unclear.

In this present work, the effect of Y content (0.1, 0.2, 0.4 wt.%) on the precipitation behavior, oxidation and mechanical properties of the as-cast Ti-Al-Sn-Zr-Mo-Si-W-Nb series high-temperature titanium alloys were systematically investigated. In addition, the corresponding oxidation and high-temperature fracture mechanism were also discussed.

## 2. Materials and Methods

Ti-5Al-2.75Sn-3Zr-1.5Mo-0.45Si-1W-2Nb-xY (x = 0.1, 0.2, 0.4) alloys, developed for the application at 700 °C based on traditional design methods for near α titanium alloys, were prepared by the vacuum electromagnetic levitation melting technique. Pure Ti, Y, Al, Zr, Sn, Mo, Nb and Si were added in the form of elemental alloys, and W was added in the form of a Ti-90W master alloy. Melting stocks weighing 1 kg were melted in a water-cooled copper crucible. Before melting, the sealed evacuated chamber was washed three times with 99.999% high-purity argon, and the internal atmosphere environment was controlled below 10^−3^ Pa. The melting process was at a dry high-purity argon (99.999%) atmosphere maintained at 5 × 10^3^ Pa. After the raw materials were melted uniformly, the melt was kept for 2 min and then cooled to room temperature in the crucible. Each ingot was melted four times to keep the composition homogeneity. The actual compositions of the prepared titanium alloys were analyzed by an X-ray fluorescent spectrometer (XRF, Panalytical, Almelo, The Netherland), and the results were given in Table 1.

The specimens for microstructure characterization, oxidation and mechanical performance tests were cut by wire-cut electrical discharge machining (WEDM). The phases and oxidation products of the as-cast alloys were characterized by a Smartlab (9 kW) X-ray diffractometer (XRD, Rigaku, Akishima, Japan) with a scanning speed of 10°/min. The microstructure, oxidation morphology and tensile fracture of the alloys were observed by the Quanta 450-FEG type scanning electron microscope (SEM, FEI, Eindhoven, The Netherland) equipped with an energy dispersive X-ray spectrometer (EDS, FEI, Eindhoven, The Netherland). The precipitates were characterized by a Tecnai G2 F30 transmission electron microscope (TEM, FEI, Amsterdam, The Netherland). The oxidation mechanisms were analyzed by LabRAM HR Evolution type laser Raman (LRS, HORIBA, Kyoto, Japan) and ESCALAB 250 Xi X-ray photoelectron spectroscopy (XPS, TMO, NY, USA). Specimens for SEM observations were etched with Kroll’s reagent (HF: HNO_3_: H_2_O = 1: 3: 7, Vol.%). TEM observation specimens were ground to 40 μm and then punched to Φ 3 mm discs for ion thinning.

The high-temperature oxidation resistance test was carried out in a KSL-1100X type medium temperature box resistance furnace (HF-Kejing, Hefei, China) at 650 °C, 700 °C and 750 °C. The dimensions of the oxidation specimen were 10 × 10 × 3 mm^3^. Each specimen was measured by a vernier caliper, and the surface area was recorded. The specimen for the oxidation resistance test was placed in a dried corundum crucible, and the weight of the specimen and crucible was measured by an electronic balance with an accuracy of 0.01 mg as the initial weight before the test. The whole oxidation test lasted for 120 h, and the crucible was taken out every 12 h to obtain the weight changes. Three specimens were placed in each group to calculate the average weight gain value to improve the measurement accuracy.

**Figure 1:**
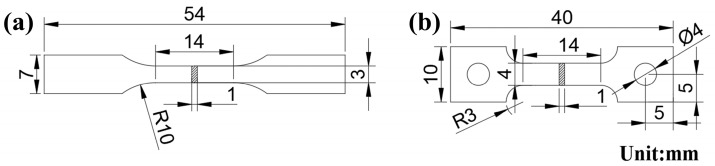
Geometric dimension of the specimens for (**a**) room-temperature tensile test; and (**b**) high-temperature tensile test.

The room-temperature tensile test was implemented on an Instron-5848 (Boston, MA, USA) tensile testing machine at a tensile rate of 1 mm/min. According to the ASTM E8/E8M-16 standard, a 15 mm gauge length extensometer was used to measure the strain to exclude the influence of the elastic strain of the grips. The high-temperature tensile test was carried out on the CMT-5205 tensile testing machine in the air environment at 700 °C and 750 °C. The tensile rate was 0.5 mm/min. The tensile test was repeated three times under each condition. The dimensions of the tensile specimens for room- and high-temperature tests are shown in Figure 1.

## 3. Results and Discussion

### 3.1. Microstructure

**Figure 2:**
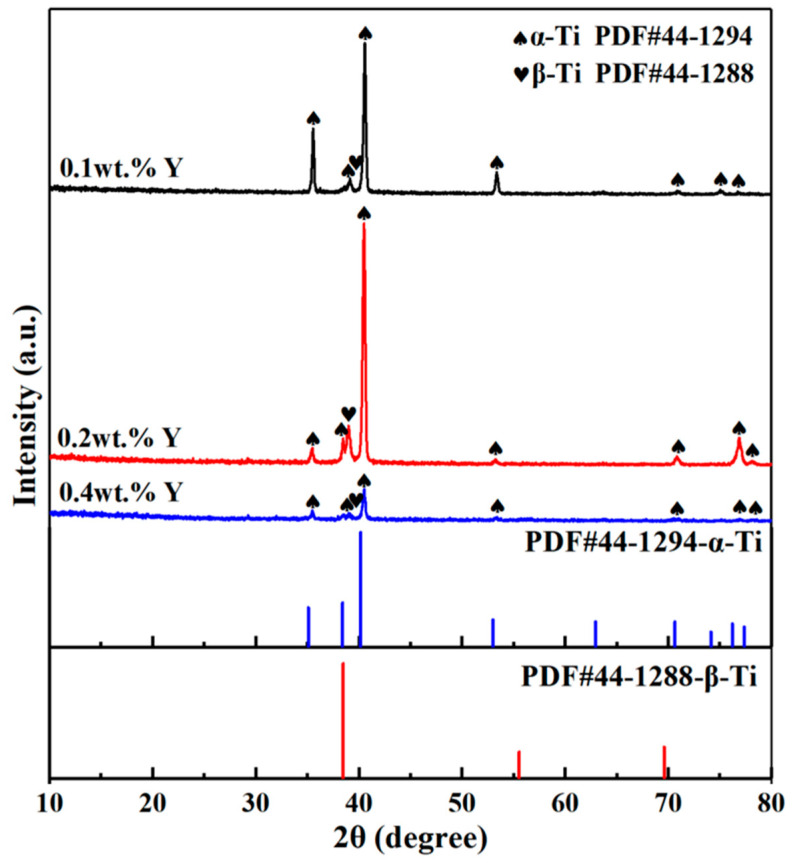
XRD patterns of as-cast titanium alloys with different Y content.

Figure 2 shows the XRD spectra of the as-cast Ti-5Al-2.75Sn-3Zr-1.5Mo-0.45Si-1W-2Nb-xY (x = 0.1, 0.2, 0.4) alloys. It can be seen that the as-cast alloys are mainly composed of close-packed hexagonal (hcp) α-Ti phase and a small amount of body-centered cubic (bcc) β-Ti phase, and all the alloys still belong to near-α titanium alloy. It also can be observed that the increase of Y content promotes the diffraction peak of the α-Ti phase to move to a low angle direction. According to the Bragg’s equation [11], the shift of the diffraction peak to a low angle is caused by lattice expansion. The lattice parameters of the α-Ti phase in 0.1 wt.%, 0.2 wt.% and 0.4 wt.% Y composition alloys were obtained by XRD refinement, and the averages of three measurement results are a = 0.2914 nm, c = 0.4643 nm; a = 0.2923 nm, c = 0.4678 nm; and a = 0.2920 nm, c = 0.4687 nm, respectively. It can be seen that both the lattice parameter “a” and “c” increase due to the Y with large atomic radius substituting for Ti atoms with small atomic radius, with the increase of Y content.

**Figure 3:**
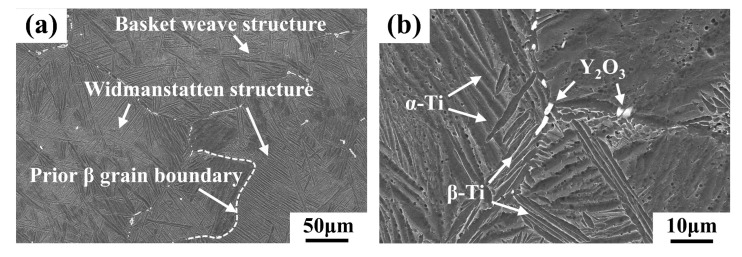

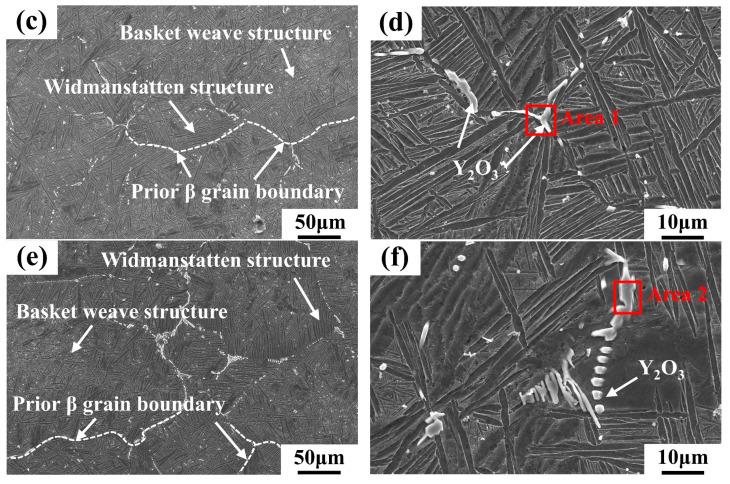
SEM microstructures of the as-cast alloys with different Y content: (**a**,**b**) 0.1 wt.% Y; (**c**,**d**) 0.2 wt.% Y; (**e**,**f**) 0.4 wt.% Y.

Figure 3 shows the SEM microstructures of the as-cast alloys with different Y content. It can be seen that all the microstructures are pf the widmanstätten structure, with typical basket weave features. The prior β grains in the microstructure are relatively coarse, and they are composed of lamellar α-Ti and residual β-Ti phases. The prior β grain size and α lamellar spacing decrease with the increase of Y content. The addition of the Y element can improve the energy state of the structure; thus, the grains in the microstructures have more growth orientations, resulting in a typical basket weave feature. It also can be observed that some white precipitates are distributed at the prior β grain boundaries. As the Y content increases, the morphologies of the precipitates change from short rod to long strip, and they gradually form a network distribution along the prior β grain boundaries. The number of precipitates increases with the increase of Y content. According to the EDS results presented in Table 2, it can be determined that the precipitates are Y_2_O_3_ phase.

**Figure 4:**
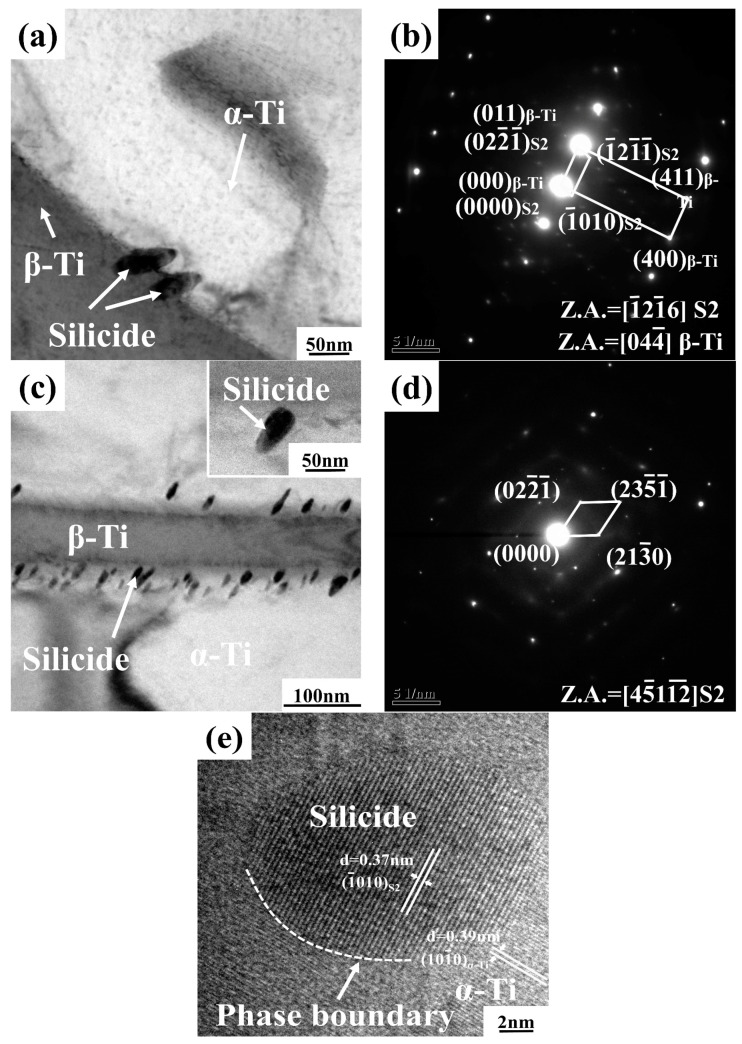
(**a**) Bright field TEM image of the as-cast alloy with 0.1 wt.% Y; (**b**) SAED pattern of the silicide taken from (**a**); (**c**) Bright field TEM image of the as-cast alloy with 0.2 wt.% Y; (**d**) SAED pattern of the silicide taken from (**c**); (**e**) HRTEM image of silicide in as-cast alloy with 0.1 wt.% Y.

Figure 4 shows the TEM images of the alloys with Y content of 0.1 wt.% and 0.2 wt.%. It can be seen that the ellipsoidal precipitates with an average size of about 20–60 nm distributed near the β phases (Figure 4a,c) [18,19]. According to the selected area electron diffraction (SAED) analysis presented in Figure 4b,d, the precipitated phase is hexagonal (TiZr)_6_Si_3_ silicide (S_2_, a = 0.701 nm, c = 0.370 nm). The addition of the Y element in the alloys will reduce the solid solubility of Si in the β phases and promote the precipitation of silicides. It also can be seen that the (TiZr)_6_Si_3_ silicide has an orientation relationship with the β-Ti phase: (02$\bar{2}$ $\bar{1}$)_(TiZr)_6_Si_3__ // (011)_β-Ti_, [$\bar{1}$2$\bar{1}$6]_(TiZr)_6_Si_3__ // [04$\bar{4}$]_β-Ti_ (Figure 4b). Figure 4e shows the high-resolution transmission electron microscopy (HRTEM) image of the interfacial structure between the (TiZr)_6_Si_3_ silicide and α-Ti phase [20,21]. It can be seen that the interface between the (TiZr)_6_Si_3_ silicide and α-Ti phase is clean, without defects, and it belongs to a semi-coherent relationship.

### 3.2. Y_2_O_3_ Precipitation

**Figure 5:**
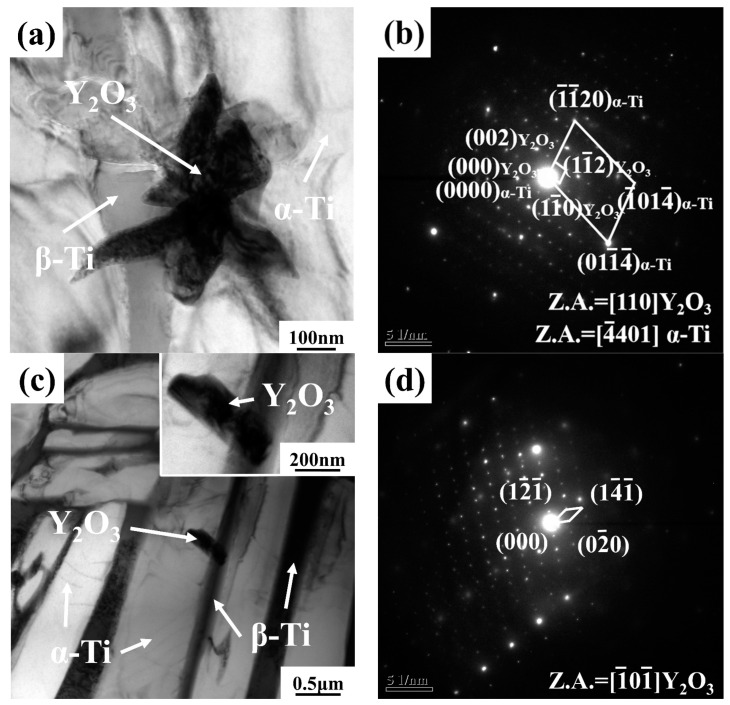
(**a**) Bright field TEM image of the Y_2_O_3_ in the as-cast alloy with 0.1 wt.% Y; (**b**) SAED pattern of the Y_2_O_3_ taken from (**a**); (**c**) Bright field TEM image of the Y_2_O_3_ in the as-cast alloy with 0.4 wt.% Y; (**d**) SAED pattern of the Y_2_O_3_ taken from (**c**).

Figure 5 shows the TEM images of the as-cast alloys with 0.1 wt.% and 0.4 wt.% Y content. It can be seen that the massive particles, with a size of about 300–400 nm, precipitated from the β phase (Figure 5a,c). They can be indexed as Y_2_O_3_ phase according to the SAED analyses (Figure 5b,d). The orientation relationships between Y_2_O_3_ phase and α-Ti phase in 0.1 wt.% Y composition alloy are established as: (002)_Y_2_O_3__ // ($\bar{1}$$\bar{1}$20)_α-Ti_, [110]_Y_2_O_3__ // [$\bar{4}$401]_α-Ti_.

**Figure 6:**
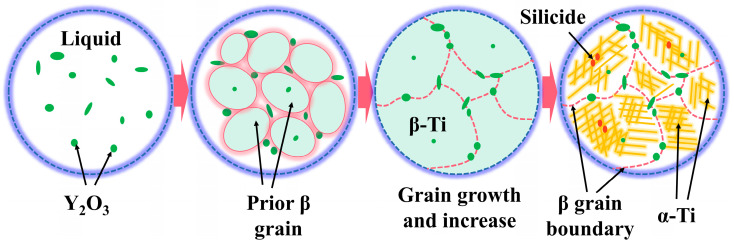
Schematic view of solidification path of the high-temperature titanium alloy containing Y.

The solid solubility of the Y element in titanium alloy is low, and it has strong chemical activity and deoxidation, which can capture the interstitial oxygen in the Ti matrix to form the Y_2_O_3_ phase [22]. Figure 6 sketches the possible solidification path of the high-temperature titanium alloys containing Y. It can be seen that the Y_2_O_3_ phase can be directly formed in the liquid phase due to its higher melting point than the titanium alloy. As the solidification temperature decreases, the β phase forms, and some Y_2_O_3_ phases can serve as a heterogeneous nucleation site for the β phase, improving its nucleation rate. In this case, the Y_2_O_3_ phase in the remaining liquid phase will be pushed into the interdendritic region of the β phase. Therefore, most Y_2_O_3_ phases would eventually be distributed at the prior β grain boundaries. As the temperature continues to decrease, the silicide phase will precipitate from the β phase; then, β/α transformation occurs. Thus, the Y_2_O_3_ phase can cooperate with the silicide phase to produce a pinning effect on grain boundaries and improve the mechanical properties of the alloy at high temperatures.

### 3.3. Oxidation Resistance

**Figure 7:**
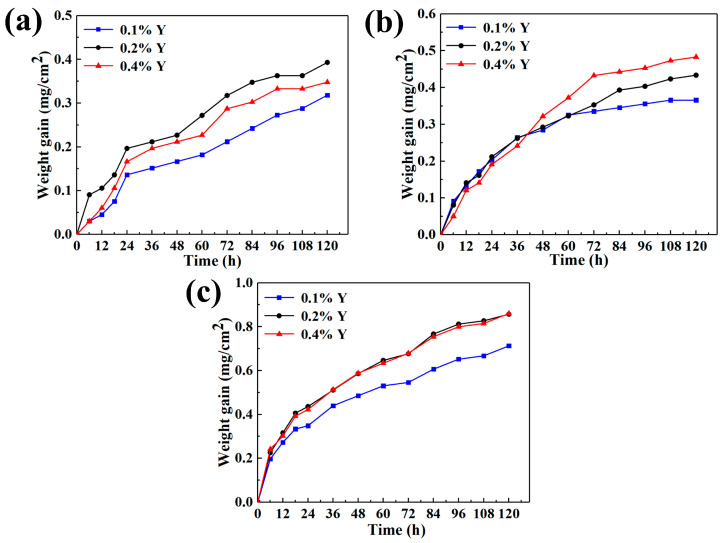
Oxidation weight gain curves of as-cast alloys with different Y content varying with oxidation time at different temperatures: (**a**) 650 °C; (**b**) 700 °C; (**c**) 750 °C.

Figure 7 illustrates the oxidation weight gain curves of as-cast alloys with different Y content, oxidized at different temperatures for up to 120 h. It can be seen that the oxidation weight gain of alloys with different Y content increases with the increase of oxidation time. The oxidation weight gain of alloys increases with the increase of oxidation temperature at the same Y content. Further increase in Y content under the same conditions has adverse effects on the oxidation resistance of the alloy. The oxidation weight gain per unit area of as-cast alloys with different Y content at different temperatures for 120 h is shown in Table 3. Combined with the analysis in Figure 7, the 0.1 wt.% Y composition alloy has the smallest weight gain value and the best high-temperature oxidation resistance.

The oxidation kinetics can be expressed by Equation (1) [23]:

∆*W^n^ = k_n_t* (1)

where ΔW is the oxidation weight gain (mg/cm^2^), n is the reaction index, k_n_ is the oxidation reaction rate constant, and t is the oxidation time (s). Equation (1) is subjected to natural logarithm processing to obtain Equation (2):

*nlg(*∆*W) = lgk_n_ + lgt* (2)

This demonstrates that there is a linear relationship between lg(∆W) and lgt, and n and k_n_ can be used as parameters to characterize the oxidation behaviors of materials. The fitting parameters of the alloys at each oxidation temperature are provided in Table 4. It can be seen that the oxidation reaction index n of the alloy is between 1 and 2, which shows that the oxidation of the alloys is controlled by diffusion process [24,25] and the oxidation weight gain of the alloy follows the linear-parabolic law.

**Figure 8:**
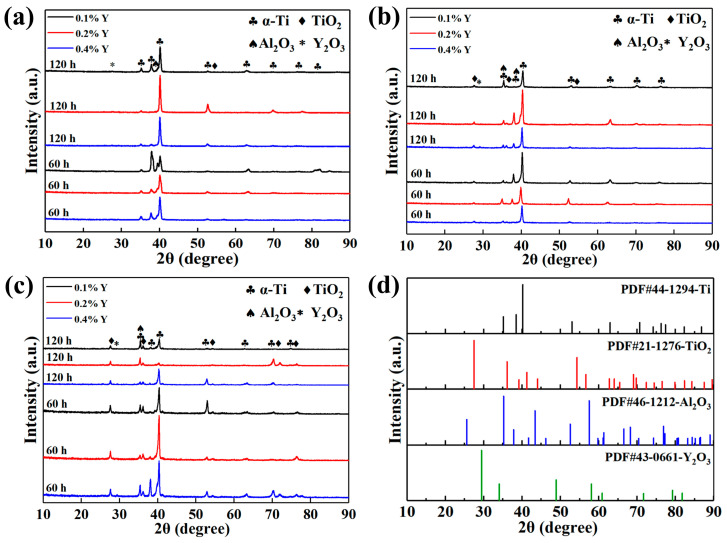
XRD patterns of the as-cast alloys with different Y content after being oxidized at different temperatures for 60 h and 120 h: (**a**) 650 °C; (**b**) 700 °C; (**c**) 750 °C; (**d**) standard spectrum.

Figure 8 shows the XRD patterns of the as-cast alloys with different Y content after being oxidized at different temperatures for 60 h and 120 h. It can be seen that the oxidation products of the as-cast alloys are mainly rutile-TiO_2_, Al_2_O_3_ and Y_2_O_3_. The lattice constants of α-Ti after oxidation were calculated according to the XRD data, and the results are exhibited in Table 5. It can be seen that both the lattice constant ‘a’ and ‘c’ of the as-cast alloys after oxidation increase, which is caused by the entry of small-sized oxygen atoms into the lattice gap during the oxidation process [26,27]. With the increase of oxidation temperature and time, some interstitial oxygen in the lattice is consumed form oxidation products. Thus, the degree of lattice expansion will be reduced.

**Figure 9:**
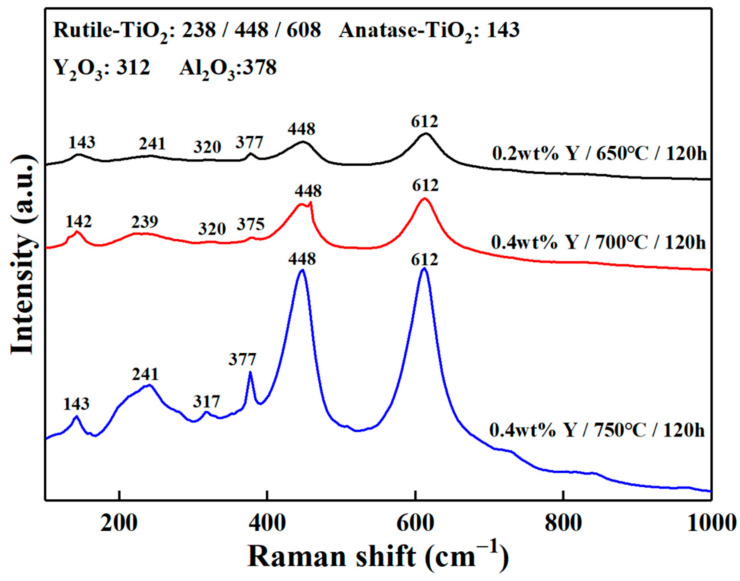
Laser Raman spectra of 0.2 wt.% Y composition alloy oxidized at 650 °C for 120 h and 0.4 wt.% Y composition alloy oxidized at 700 °C and 750 °C for 120 h.

To further analyze the compositions of the surface oxidation products, LRS and XPS tests were conducted. The 0.2 wt.% Y composition alloy oxidized at 650 °C for 120 h and 0.4 wt.% Y composition alloy oxidized at 700 °C and 750 °C for 120 h were selected for the laser Raman test in the range of 100~1000 cm^−1^. The corresponding laser Raman spectra are displayed in Figure 9. It can be seen that the oxidation products are composed of rutile-TiO_2_ (238 cm^−1^, 448 cm^−1^, 608 cm^−1^), anatase-TiO_2_ (143 cm^−1^), Y_2_O_3_ (312 cm^−1^) and Al_2_O_3_ (378 cm^−1^) [28]. However, anatase-TiO_2_ was not found in the XRD analysis (Figure 8) due to its low content and easy encapsulation by rutile-TiO_2_.

**Figure 10:**
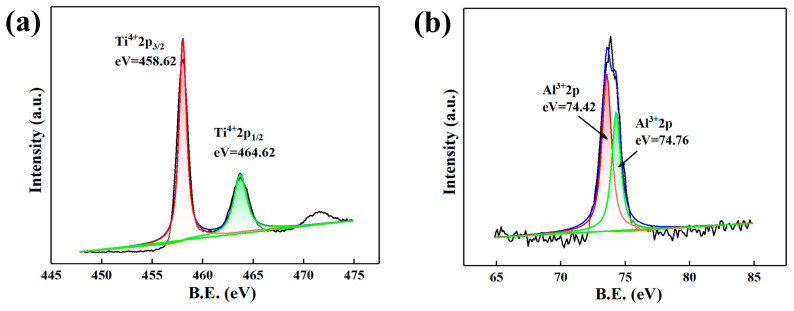

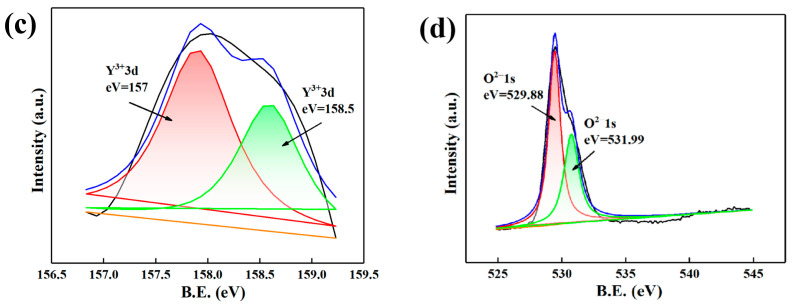
XPS spectra of 0.4 wt.% Y composition alloy oxidized at 750 °C for 120 h: (**a**) Ti2p; (**b**) Al2p; (**c**) Y3d; (**d**) O1s.

Figure 10 illustrates the XPS spectra of 0.4 wt.% Y composition alloy oxidized at 750 °C for 120 h. It can be seen that the valence states of elements in the oxidation products are Ti^4+^ (458.62 eV, 464.62 eV), Al^3+^ (74.76 eV, 74.42 eV), O^2−^ (529.88 eV, 531.99 eV) and Y^3+^ (158.5 eV, 156.47 eV, 157.0 eV) [29]. Among them, O^2−^ with a binding energy of 529.88 eV and 531.99 eV is oxygen in titanium oxide and alumina, respectively. Ti^4 +^ with a binding energy of 458.62 eV and 464.62 eV is titanium in anatase-TiO_2_ and rutile-TiO_2_, respectively. The fitting results of XPS are consistent with the results of laser Raman and XRD analysis. Therefore, it can be considered that the oxidation products of the alloy are composed of rutile-TiO_2_, anatase-TiO_2_, Y_2_O_3_ and Al_2_O_3_.

**Figure 11:**
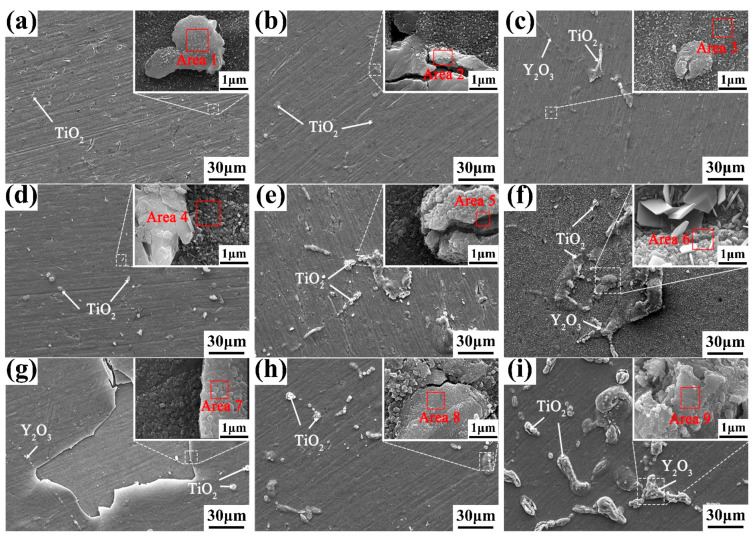
Surface morphology of the (**a**,**d**,**g**) 0.1 wt.% Y; (**b**,**e**,**h**) 0.2 wt.% Y; and (**c**,**f**,**i**) 0.4 wt.% Y alloys oxidized at (**a**–**c**) 650 °C; (**d**–**f**) 700 °C; and (**g**–**i**) 750 °C for 120 h.

Figure 11 shows the surface morphology of as-cast alloys with different Y content after oxidation for 120 h at different temperatures. EDS analysis results of the oxidation surface marked in Figure 11 are listed in Table 6. It can be seen that the oxidation surfaces are mainly composed of granular TiO_2_, short rod Y_2_O_3_ and Al_2_O_3_. The oxide particles on the surface of the alloys oxidized at 650 °C are smaller, and no serious cracks are observed on the oxidized surfaces. As the oxidation temperature exceeds 700 °C, the amount and size of the oxide particles increases significantly. Therefore, stress would occur between the oxide particles and the matrix, causing cracks. These cracks provide a large number of channels for the internal diffusion of oxygen atoms, which is not conducive to the formation of a uniform and dense oxide film. When the oxidation temperature exceeds 750 °C, the integrity of the oxide layer is seriously damaged. Thus, the oxidation resistance of the alloy decreases with the increase of oxidation temperatures. The number and size of oxidation products increase with the increase of Y content, further indicating that the 0.1 wt.% Y composition alloy has the best high-temperature oxidation resistance.

**Figure 12:**
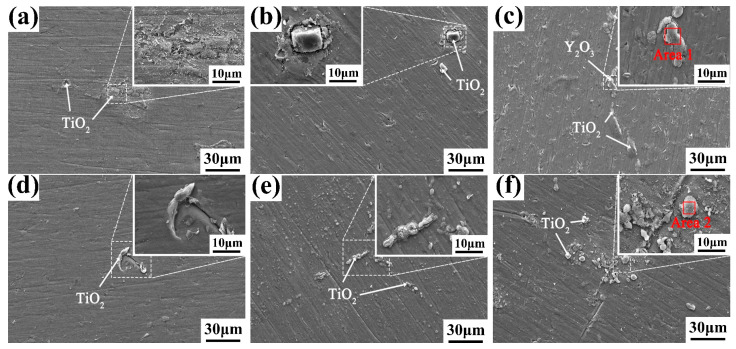

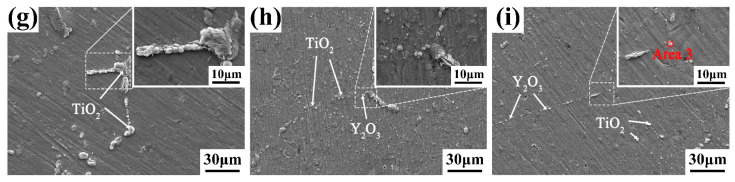
Surface morphology of the (**a**,**d**,**g**) 0.1 wt.% Y; (**b**,**e**,**h**) 0.2 wt.% Y; and (**c**,**f,i**) 0.4 wt.% Y alloys oxidized at (**a**–**c**) 650 °C; (**d**–**f**) 700 °C; and (**g**–**i**) 750 °C for 60 h.

To further explore the change process of the oxide layer in different oxidation stages, the surface morphologies of as-cast alloys with different Y content after oxidation for 60 h at different temperatures were characterized, and the results are presented in Figure 12. EDS analysis results of the oxidation surface marked in Figure 12 are listed in Table 7. It can be seen that the morphology of the surface oxide particles under 60 h and 120 h oxidation is similar; however, there are fewer surface cracks and oxide spalling under 60 h oxidation. This indicates that the external oxide layer has been basically formed after 60 h of oxidation. After 60 h, the oxidation mainly enters the internal diffusion stage of O atoms, which can damage the density and integrity of the oxide film.

To further study the composition of oxidation products, thermodynamic analysis was carried out. The oxidation reaction of titanium alloy follows the Vant Hoff isothermal equation, as specified in Equation (3) [30]:

∆*G* = ∆*G*_0_ − *RTlnP*_*O*_2__ (3)

where ΔG is the Gibbs free energy change, ΔG_0_ is the standard free energy change. R is the gas constant, T is the Kelvin temperature, and P_O2_ is the equilibrium oxygen partial pressure. As described above, TiO_2_ and Al_2_O_3_ are the main oxidation products. The ΔG of TiO_2_ and Al_2_O_3_ at 650 °C, 700 °C and 750 °C is calculated according to Equation (3), and the results are listed in Table 8. It can be seen that Al_2_O_3_ and TiO_2_ can coexist on the oxide surface due to their ΔG value being negative. The stability of Al_2_O_3_ is also higher than that of TiO_2_. However, TiO_2_ was easier to form due to the much higher content of Ti than Al.

**Figure 13:**
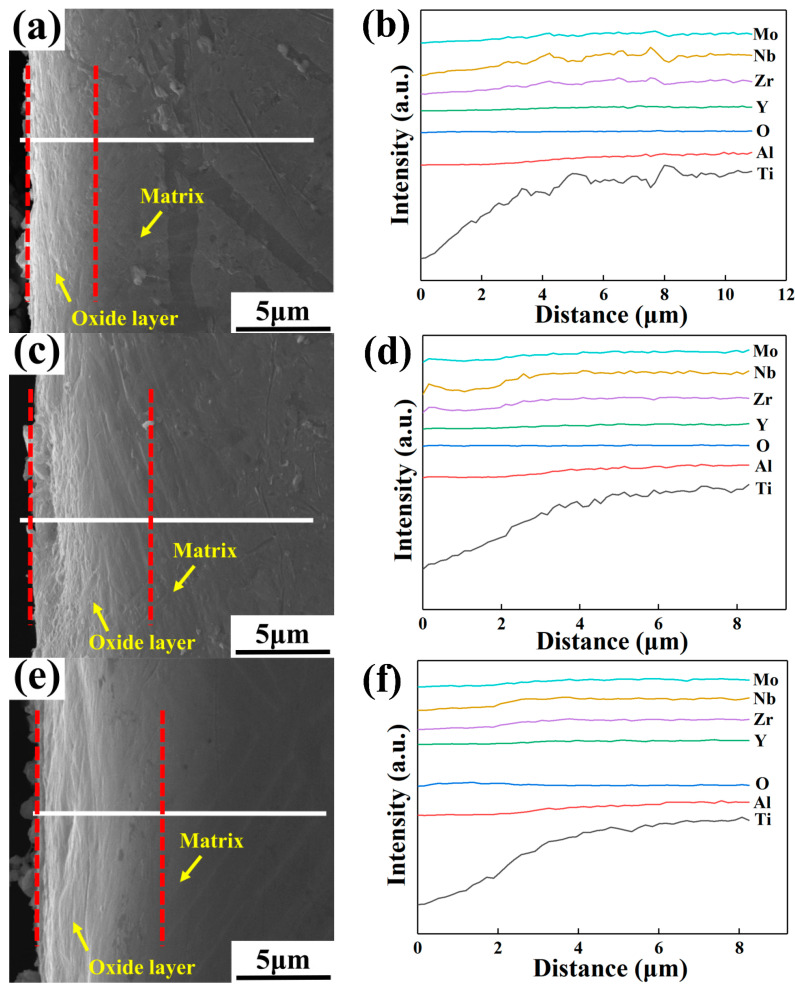
Cross-section morphologies and elemental distribution curves of the oxidation surfaces of as-cast alloys with different Y content after oxidation for 120 h at 650 °C: (**a**,**b**) 0.1 wt.% Y; (**c**,**d**) 0.2 wt.% Y; (**e**,**f**) 0.4 wt.% Y.

**Figure 14:**
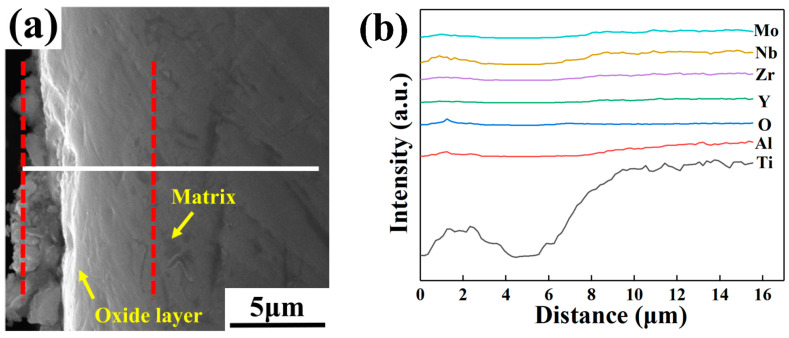

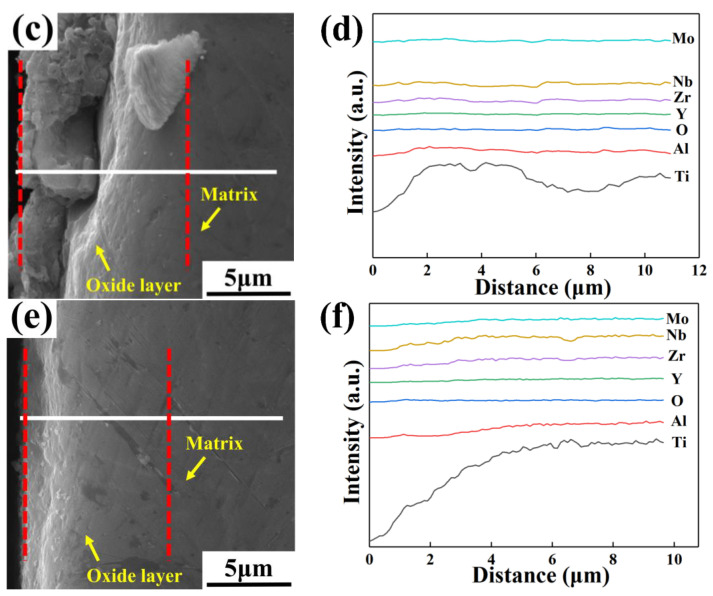
Cross-section morphologies and elemental distribution curves of the oxidation surfaces of as-cast alloys with different Y content after oxidation for 120 h at 700 °C: (**a**,**b**) 0.1 wt.% Y; (**c**,**d**) 0.2 wt.% Y; (**e**,**f**) 0.4 wt.% Y.

**Figure 15:**
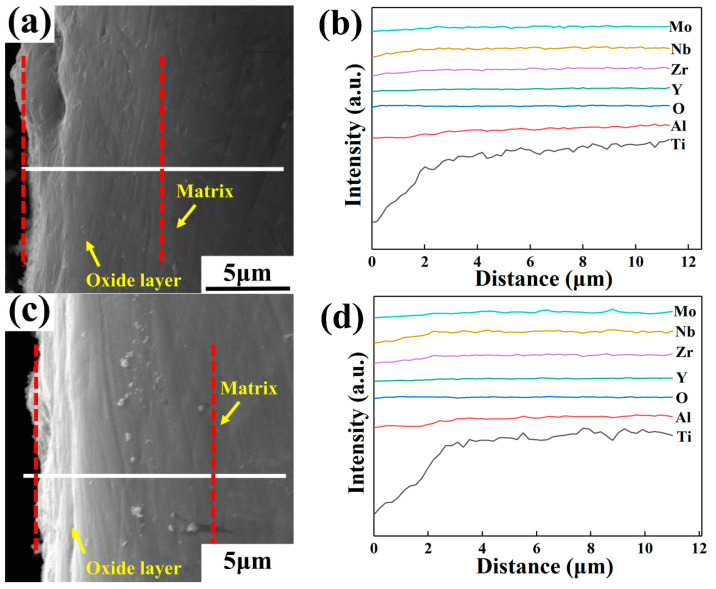

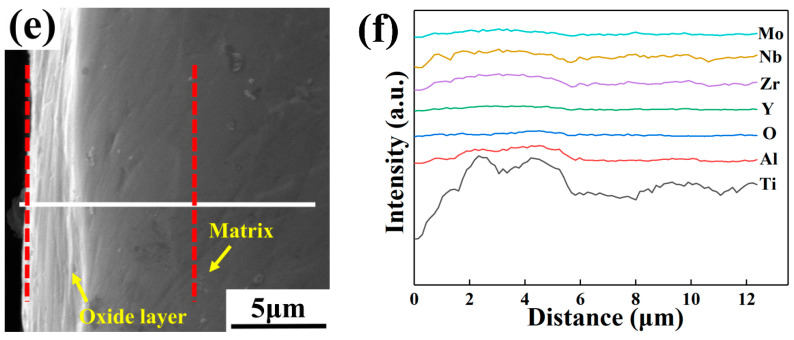
Cross-section morphologies and elemental distribution curves of the oxidation surfaces of as-cast alloys with different Y content after oxidation for 120 h at 750 °C: (**a**,**b**) 0.1 wt.% Y; (**c**,**d**) 0.2 wt.% Y; (**e**,**f**) 0.4 wt.% Y.

Figure 13, Figure 14 and Figure 15 show the cross-section morphologies and elemental distribution curves of the oxidation surfaces of as-cast alloys with different Y content for different temperatures under oxidation for 120 h. It can be seen that the oxidation profile can be divided into the surface oxide film, oxide layer and matrix. The surface oxide film is difficult to distinguish due to its extremely thin thickness. The thickness of the oxide layer of the alloys with different Y content oxidized at different temperatures for 120 h is illustrated in Table 9. It can be seen that under the same oxidation temperature conditions, the 0.1 wt.% Y composition alloy has the smallest oxide layer thickness, further proving that it has the best oxidation resistance. According to the elemental distribution line scan and thermodynamic calculations analysis, it can be seen that Al_2_O_3_ is mainly distributed in the outermost layer of the oxide film, the inner layer of the oxide film is mainly composed of TiO_2_ and a small amount of Al_2_O_3_, and the oxide layer is mainly TiO_2_. This shows that in the early stage of oxidation, Ti and Al elements diffuse outward and oxidation reaction occurs; the generated oxides cover Y_2_O_3_ to form an oxide film with a loose structure. With the extension of oxidation time, oxygen atoms can diffuse inward through the gaps and cracks in the oxide film and oxidize with Ti atoms inside the matrix to form an oxygen-rich layer. Due to the different types of oxidation products in the oxide surface and oxide layer, the stress concentration generated means that the oxide film and the oxide layer cannot be tightly bonded, resulting in the oxide layer being peeled off at more than 750 °C.

### 3.4. Mechanical Properties

**Figure 16:**
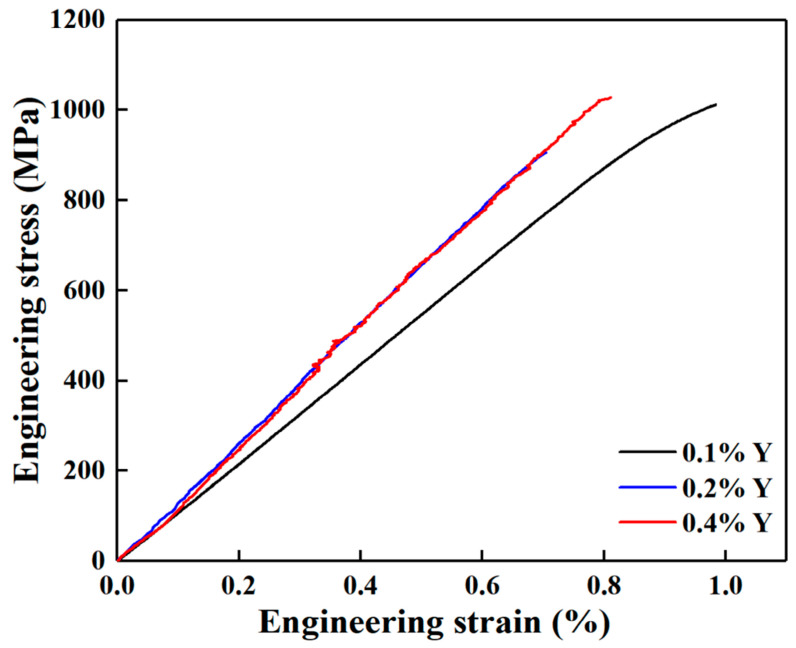
Room-temperature tensile stress–strain curves of as-cast alloys with different Y content.

**Figure 17:**
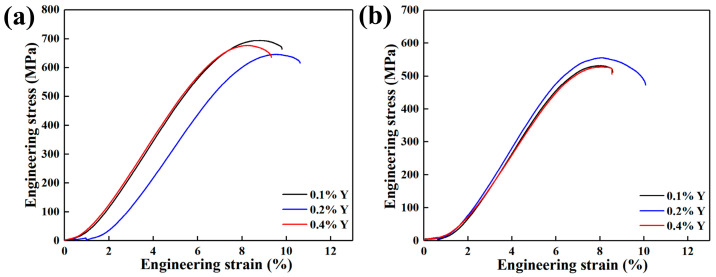
High-temperature tensile stress–strain curves of as-cast alloys with different Y content under different test temperatures: (**a**) 700 °C; (**b**) 750 °C.

Figure 16 shows the room-temperature tensile engineering stress–strain curves of the alloys with different Y content, and Table 10 lists the corresponding values of the mechanical properties. It can be seen that the elongation of the cast alloys are all less than 1%, and the engineering stress–strain curves of the alloys at room temperature are all close to straight lines, indicating that the material behavior can be considered as brittle. The ultimate tensile strength (UTS) of the alloys decreases first and then increases with the increase of Y content. The alloy with 0.1 wt.% Y content has the best comprehensive mechanical properties at room temperature, and the UTS and elongation (EL) are 1012 MPa and 1.0 %, respectively. Figure 17 shows the high-temperature tensile stress–strain curves of the alloys with different Y content. Combined with the data in Table 10, it can be seen that the UTS of the alloys at 700 °C decreases first and then increases with the increase of Y content, whereas the elongation shows an opposite trend. The UTS and elongation of the alloy with 0.1 wt.% Y is 694 MPa and 9.8%, showing an optimal comprehensive performance. The UTS and elongation of the alloys at 750 °C increase first and then decrease with the increase of Y content. The UTS and elongation of the alloy with 0.2 wt.% Y content are 556 MPa and 10.1%, which is 4.5%, 17.4% and 5.3%, 17.4% higher than that of the alloy with 0.1 wt.% and 0.4 wt.% Y content, respectively.

**Figure 18:**
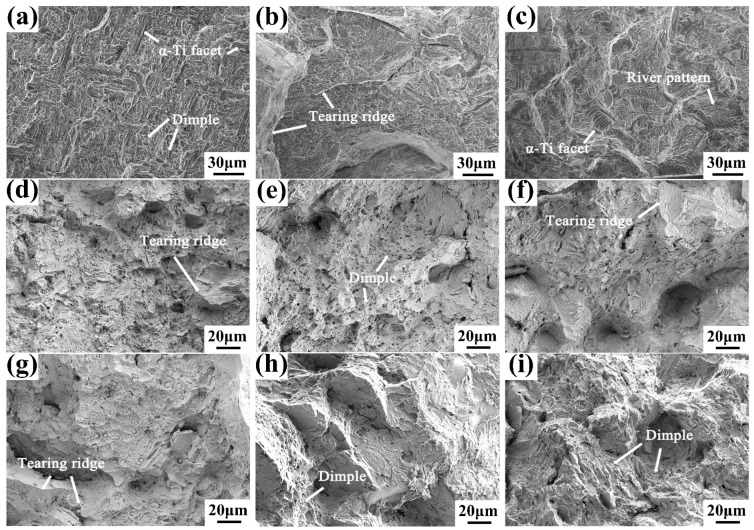
SEM images of tensile fracture of (**a**,**d**,**g**) 0.1 wt.% Y; (**b**,**e**,**h**) 0.2 wt.% Y; and (**c**,**f**,**i**) 0.4 wt.% Y alloys at (**a**–**c**) room temperature, (**d**–**f**) 700 °C and (**g**–**i**) 750 °C high temperatures.

Figure 18 shows the tensile fracture features of the as-cast alloys with different Y content at room temperature and high temperature. It can be seen that the fracture features of as-cast alloys at room temperature are mainly composed of larger cleavage planes and tearing edges, which are typical cleavage fracture modes, corresponding to brittle fracture. At high temperature, the fracture morphologies of as-cast alloys are the combination of number of dimples and partial tearing ridges, indicating that the plasticity of the alloy has been greatly improved.

The size and distribution of the precipitated phases will affect the mechanical properties of the alloys [31,32]. As described above, the size of Y_2_O_3_ in 0.1 wt.% Y composition alloy is small and dispersed, which has a certain dispersion strengthening effect in the alloy. With the increase of Y content, the size of Y_2_O_3_ increases, and a large-size brittle particle phase is formed in the local position. When the distribution of large-sized precipitates is uneven, it leads to more severe stress concentration and reduces strength during the tensile deformation of the alloy. This is also the reason for the large-size cleavage planes and tearing ridges in the 0.2 wt.% Y composition alloy. When the Y content increases to 0.4 wt.%, Y_2_O_3_ will be distributed more uniformly. At this time, Y_2_O_3_ forms a network structure distributed along the prior β grain boundary, which can improve the ability of the prior β grain boundary to hinder deformation and significantly reduce the weakening degree of the grain boundaries at high temperatures.

**Figure 19:**
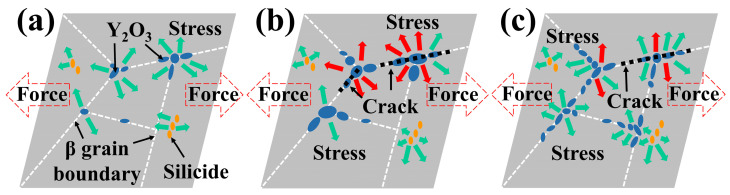
Schematic illustration of the fracture mechanism of the alloys with different Y content: (**a**) 0.1 wt.% Y; (**b**) 0.2 wt.% Y; (**c**) 0.4 wt.% Y.

In addition, the silicide in the microstructure will change the stress at the interface of α/β laths [33,34], and it synergistically affects the fracture strength and toughness of the alloy with the Y_2_O_3_ phase. Therefore, the mechanical properties of the alloys are affected by the distribution of the Y_2_O_3_ phase, silicide and temperature factors. A schematic illustration of the fracture mechanism of the alloys with different Y content is shown in Figure 19.

## 4. Conclusions

(1) The as-cast alloys with different Y content are mainly composed of α-Ti phase and a small amount of β-Ti phase. With the increase of Y content, both the α-Ti lattice parameters “a” and “c” increase.
(2) The microstructures of the alloys are all of the widmanstätten structure, with typical basket weave features. The prior β grain size and α lamellar spacing decrease with the increase of the Y content.
(3) With the increase of the Y content, the number of Y_2_O_3_ phases increases, and the morphologies of the Y_2_O_3_ phases change from short rod to long strip. The orientation relationships between the Y_2_O_3_ phase and α-Ti phase in 0.1 wt.% Y composition alloy are established as: (002)_Y_2_O_3__ // ($\bar{1}$ $\bar{1}$20)_α-Ti_, [110]_Y_2_O_3__ // [ $\bar{4}$401]_α-Ti_.
(4) (TiZr)_6_Si_3_ silicide is precipitated from the β-Ti phase. It has an orientation relationship with the β-Ti phase: (02 $\bar{2}$ $\bar{1}$)_(TiZr)_6_Si_3__ // (011)_β-Ti_, [ $\bar{1}$2 $\bar{1}$6]_(TiZr)_6_Si_3__ // [04 $\bar{4}$]_β-Ti_.
(5) The oxidation resistance of the alloys decreases with the increase of oxidation temperature and time. The 0.1 wt.% Y composition alloy has the best high-temperature oxidation resistance at different temperatures. The oxidation behaviors of the alloys conform to the linear-parabolic law, and the oxidation products of the alloy are composed of rutile-TiO_2_, anatase-TiO_2_, Y_2_O_3_ and Al_2_O_3_.
(6) The room temperature UTS of the alloys decreases first and then increases with the increase of Y content; the 0.1 wt.% Y composition alloy has best room temperature mechanical properties, with a UTS of 1012 MPa and elongation of 1.0%. The UTS of the alloys at 700 °C decreases first and then increases with the increase of Y content, whereas the elongation shows an opposite trend. The UTS and elongation of the alloy with 0.1 wt.% Y is 694 MPa and 9.8%, showing an optimal comprehensive performance. The UTS and elongation of the alloys at 750 °C increase first and then decrease with the increase of Y content. The optimal UTS and elongation of the alloy is 556 MPa and 10.1%, obtained in 0.2 wt.% Y composition alloy. The cleavage and dimple fractures are the primary fracture mode for the room- and high-temperature tensile fracture, respectively.

## Figures and Tables

**Table 1:** Actual chemical compositions of as-cast Ti-5Al-2.75Sn-3Zr-1.5Mo-0.45Si-1W-2Nb-xY alloys (wt.%).

Alloys	Al	Sn	Zr	Mo	Si	W	Nb	Y	Ti
0.1 wt.% Y	4.54	2.57	2.73	1.40	0.42	1.09	2.05	0.13	Bal.
0.2 wt.% Y	4.67	2.89	2.85	1.47	0.46	1.07	2.07	0.28	Bal.
0.4 wt.% Y	4.58	2.90	2.87	1.48	0.52	1.14	2.07	0.39	Bal.

**Table 2:** EDS analysis results of areas 1 and 2 marked in Figure 3 (at.%).

Position	Ti	Al	Y	O	Nb	Sn	Zr	Mo	Si	W
Area 1	1.75	0.18	40.75	57.14	0.13	0.05	-	-	-	-
Area 2	2.45	0.33	37.19	55.32	0.07	0.20	3.25	0.18	0.26	0.75

**Table 3:** Oxidation weight gain per unit area of as-cast alloys with different Y content at different temperatures for 120 h (mg/cm^2^).

Alloy	650 °C	700 °C	750 °C
0.1 wt.% Y	0.318	0.366	0.713
0.2 wt.% Y	0.393	0.433	0.857
0.4 wt.% Y	0.348	0.483	0.860

**Table 4:** The oxidation reaction index (n) and reaction rate constant (k_n_) of alloys with different Y content at different temperatures for 120 h.

Temperature/°C	Alloy	n	k_n_
650	0.1 wt.% Y	1.2933	1.99 × 10^−3^
	0.2 wt.% Y	1.9178	1.42 × 10^−3^
	0.4 wt.% Y	1.2804	2.66 × 10^−3^
700	0.1 wt.% Y	2.1187	1.28 × 10^−3^
	0.2 wt.% Y	1.8295	2.07 × 10^−3^
	0.4 wt.% Y	1.3637	3.85 × 10^−3^
750	0.1 wt.% Y	2.3956	3.62 × 10^−3^
	0.2 wt.% Y	2.2684	6.19 × 10^−3^
	0.4 wt.% Y	2.3051	5.94 × 10^−3^

**Table 5:** Lattice constant of α-Ti of the as-cast alloys with different Y content before and after being oxidized at different temperatures for 60 h and 120 h.

Alloy	Temperature/°C	a (0.1–0.2–0.4 wt.%Y)/nm	c (0.1–0.2–0.4 wt.%Y)/nm
As-cast	25	0.29136–0.29233–0.29198	0.46426–0.46780–0.46871
60 h Oxidation	650	0.29376–0.29485–0.29482	0.47020–0.47185–0.47443
	700	0.29377–0.29626–0.29473	0.47253–0.47755–0.47292
	750	0.29304–0.29350–0.29311	0.46983–0.47020–0.47068
120 h Oxidation	650	0.29415–0.29434–0.29516	0.47452–0.47568–0.47413
	700	0.29284–0.29289–0.29421	0.46932–0.47185–0.47257
	750	0.29253–0.29302–0.29397	0.46997–0.47317–0.46895

**Table 6:** EDS analysis results of the oxidation surface marked in Figure 11 (wt.%).

Position	Ti	Al	Y	O	Zr	Nb	Mo
Area 1	55.16	5.48	0.40	35.26	1.83	1.62	0.25
Area 2	35.77	4.17	6.46	45.10	3.02	5.11	0.37
Area 3	59.02	5.59	0.06	27.25	2.70	4.97	0.41
Area 4	45.59	5.52	0.02	40.73	2.13	5.89	0.12
Area 5	51.08	5.43	0.11	35.28	2.66	5.21	0.23
Area 6	43.55	5.68	0.01	44.24	2.35	3.91	0.26
Area 7	75.48	3.93	0.11	10.10	2.19	7.77	0.42
Area 8	18.14	2.50	42.30	23.72	2.76	10.19	0.39
Area 9	38.95	9.33	8.12	33.23	2.66	7.37	0.34

**Table 7:** EDS analysis results of the oxidation surface marked in Figure 12 (wt.%).

Position	Ti	Al	Y	O	Zr	Nb	Mo
Area 1	20.00	2.49	35.36	33.24	2.31	5.89	0.71
Area 2	42.50	4.15	0.29	46.27	1.93	4.59	0.27
Area 3	39.57	3.96	4.39	43.49	3.95	4.28	0.36

**Table 8:** Gibbs free energy change (ΔG) of TiO_2_ and Al_2_O_3_ at different temperatures (kJ·mol^−1^).

ΔG	650 °C	700 °C	750 °C
TiO_2_	−750	−742	−733
Al_2_O_3_	−1381	−1365	−1349

**Table 9:** The oxide layer thickness of as-cast alloys with different Y content oxidized at different temperatures for 120 h (μm).

Temperature/°C	0.1 wt.% Y	0.2 wt.% Y	0.4 wt.% Y
650	4.9	6.2	6.4
700	7.8	10.2	8.6
750	10.2	11.3	10.6

**Table 10:** Room and high-temperature mechanical properties of the as-cast alloys with different Y content.

Temperature/°C	Alloy	UTS/MPa	EL/%
25	0.1 wt.% Y	1012 ± 5.7	1.0 ± 0.05
	0.2 wt.% Y	906 ± 2.1	0.7 ± 0.03
	0.4 wt.% Y	1028 ± 3.6	0.8 ± 0.04
700	0.1 wt.% Y	694 ± 3.4	9.8 ± 0.41
	0.2 wt.% Y	646 ± 1.2	10.6 ± 0.50
	0.4 wt.% Y	676 ± 0.7	9.3 ± 0.30
750	0.1 wt.% Y	532 ± 1.3	8.6 ± 0.32
	0.2 wt.% Y	556 ± 4.5	10.1 ± 0.43
	0.4 wt.% Y	528 ± 0.7	8.6 ± 0.38

## Data Availability

The data presented in this study are available on request from the corresponding author.
